# Long-Term Results of IFRT vs. ISRT in Infradiaphragmal Fields in Aggressive Non-Hodgkins’s Lymphoma Patients—A Single Centre Experience

**DOI:** 10.3390/cancers16030649

**Published:** 2024-02-02

**Authors:** Lea Galunic Bilic, Fedor Santek, Zdravko Mitrovic, Sandra Basic-Kinda, Dino Dujmovic, Marijo Vodanovic, Inga Mandac Smoljanovic, Slobodanka Ostojic Kolonic, Ruzica Galunic Cicak, Igor Aurer

**Affiliations:** 1 Department of Oncology, University Hospital Centre Zagreb, 10000 Zagreb, Croatia; fedor.santek@mef.hr; 2 School of Medicine, University of Zagreb, 10000 Zagreb, Croatia; zdravmitrovic@gmail.com (Z.M.); slobodankaostojic260@gmail.com (S.O.K.); igor.aurer@kbc-zagreb.hr (I.A.); 3 Division of Haematology, Department of Internal Medicine, Clinical Hospital Dubrava, 10000 Zagreb, Croatia; 4 Division of Haematology, Department of Internal Medicine, University Hospital Centre Zagreb, 10000 Zagreb, Croatia; sandra.kinda@gmail.com (S.B.-K.); dujmovicdi@gmail.com (D.D.); marijo.vodanovic@gmail.com (M.V.); 5 Division of Haematology, Department of Internal Medicine, Clinical Hospital Merkur, 10000 Zagreb, Croatia; imandac@yahoo.com; 6 Department of Radiology, University Hospital Centre Zagreb, 10000 Zagreb, Croatia; rgalunic@gmail.com

**Keywords:** non-Hodgkin’s lymphoma, radiotherapy, combined modality therapy, survival, late complications

## Abstract

**Simple Summary:**

Radiotherapy plays a crucial role in managing lymphomas. Advancements in radiation oncology have resulted in smaller treatment volumes and an improved ability to avoid nearby critical tissues and organs. Additionally, radiation therapy doses have been reduced. Our retrospective study aimed to compare the efficacy and side effects of involved-field (IFRT) versus involved-site radiotherapy (ISRT) fields in infradiaphragmal aggressive non-Hodgkin lymphoma. Addressing the persistent concern of radiotherapy toxicity, our findings highlight that scaling down the treatment volume and doses maintains efficacy and local control without compromising patient outcome. Furthermore, this approach significantly reduces both acute and long-term side effects.

**Abstract:**

(1) Background: This study aimed to examine the difference in efficacy and toxicity of involved-field (IFRT) and involved-site radiotherapy (ISRT) fields in infradiaphragmal aggressive non-Hodgkin lymphoma patients. (2) Methods: In total, 140 patients with infradiaphragmal lymphoma treated between 2003 and 2020 were retrospectively evaluated. There were 69 patients (49%) treated with IFRT, and 71 (51%) patients treated with ISRT. The median dose in the IFRT group was 36 Gy, (range 4–50.4 Gy), and in the ISRT group, it was 30 Gy (range 4–48 Gy). (3) Results: The median follow-up in the IFRT group was 133 months (95% CI 109–158), and in the ISRT group, it was 48 months (95% CI 39–57). In the IFRT group, locoregional control was 67%, and in the ISRT group, 73%. The 2- and 5-year overall survival (OS) in the IFRT and ISRT groups were 79% and 69% vs. 80% and 70%, respectively (*p* = 0.711). The 2- and 5-year event-free survival (EFS) in the IFRT and ISRT groups were 73% and 68% vs. 77% and 70%, respectively (*p* = 0.575). Acute side effects occurred in 43 (31%) patients, which is more frequent in the IFRT group, 34 (39%) patients, than in the ISRT group, 9 (13%) patients, *p* > 0.01. Late toxicities occurred more often in the IFRT group of patients, (10/53) 19%, than in the ISRT group of patients, (2/37) 5%, (*p* = 0.026). (4) Conclusions: By reducing the radiotherapy volume and the doses in the treatment of infradiaphragmatic fields, treatment with significantly fewer acute and long-term side effects is possible. At the same time, efficiency and local disease control are not compromised.

## 1. Introduction

Aggressive non-Hodgkin lymphomas (aNHLs) are a heterogeneous group of potentially fatal lymphoid malignancies that pose a significant burden on global healthcare systems [1]. For the early stages of an aNHL, adjuvant radiotherapy (RT) has become the standard treatment based on the results of two large, randomised trials: ECOG (Eastern Cooperative Oncology Group) and SWOG (Southwest Oncology Group). They proved the superiority of the combined method of treatment by comparing it with the treatment of chemotherapy (Cth) alone in the early stages (I and II) of high-grade lymphoma [2,3]. In the advanced stages of the disease, chemotherapy and immunotherapy take centre stage, but RT may still play a crucial role in specific scenarios. Consolidative RT is considered in cases where residual localized disease persists post systemic therapy or when a bulky disease requires additional intervention [4,5,6,7]. This approach seeks to enhance the depth and durability of remission, particularly in anatomical sites that may be more resistant to systemic treatments [8,9]. 

Traditionally, radiation therapy fields for NHL have focused on the supradiaphragmatic regions, as these are most commonly affected [10,11]. The RT of infradiaphragmatic region, encompassing the abdomen and pelvic areas, has been relatively underexplored in the context of NHL treatment, despite its potential clinical significance. It encompasses structures such as abdominal lymph nodes and extranodal sites, making the precise targeting of RT paramount for achieving therapeutic efficacy while minimizing potential toxicities.

Radiation techniques for lymphomas have significantly changed over the last decades. Many of the historic concepts of dose and volume have been altered [12,13]. Previously used large radiation fields are, therefore, nowadays considered unacceptable. Involved-field radiotherapy (IFRT) techniques have been replaced with smaller volumes based on detectable nodal involvement at presentation (involved site/involved node radiotherapy—ISRT/INRT) [10,14,15]. Advancements in imaging technologies have improved our ability to accurately delineate infradiaphragmatic disease involvement, facilitating precise treatment planning and delivery [16]. Girinsky and colleagues [17] introduced involved-node RT (INRT), which has a more stringent definition, requiring prechemotherapy positron emission tomography (PET) in the RT treatment position. ISRT was adopted, incorporating a prechemotherapy PET, but not requiring it to be in the RT treatment position. Moreover, it utilizes computed tomography (CT) for treatment planning after chemotherapy [10,18]. Most importantly, ISRT is a different concept from IFRT. ISRT is defined based on prechemotherapy involved nodes/sites, whereas IFRT is defined on anatomical boundaries encompassing the entire lymphatic region. In most situations, these volumes may differ significantly [18,19].

In order to analyse the effect of these changes on the outcomes of NHL patients receiving radiotherapy to infradiaphragmatic sites, we analysed treatment outcomes and the survival of patients treated with 2D and 3D conformal radiotherapy (3D-CRT) using the IFRT and ISRT treatment methods applied in the last 20 years. 

## 2. Materials and Methods

### 2.1. Patient Selection

We included patients with aNHLs starting treatment with radiotherapy (RT) to infradiaphragmal fields between January 2003 and December 2020 at our institution. All of them received chemotherapy first. Demographic and clinical characteristics and follow-up data were extracted from electronic hospital records and included age, sex, type of lymphoma, number of systemic treatment lines, the response to the patient’s last systemic treatment prior to RT, relapse, survival, and toxicities. 

Indications for RT included adjuvant RT in patients with a limited-stage aggressive NHL, an initial bulky disease, extranodal involvement or partial remission at the end of systemic therapy, and salvage RT in patients failing systemic therapy.

### 2.2. Radiotherapy 

Patients treated between 2003 and 2010 were irradiated using 2D RT delivered with two opposed parallel anteroposterior fields from a Cobalt unit or a 15 MV linear accelerator in accordance with contemporary guidelines. Radiation fields were classified as abdominal (including paraaortic lymph nodes; abdomen and pelvis), “inverted Y” (including the paraaortic, iliac, and inguinal lymph nodes) or pelvic (including iliac, inguinal, and femoral lymph nodes). RT to bulky disease was applied as involved-field RT. If residual tumour remained after chemotherapy, the target volume was adjusted. If a CR was achieved after chemotherapy, the target volume included the lymph node regions of the initial bulk. The target volume of extranodal disease included the entire initially involved extralymphatic area. RT fields encompassed the Ann Arbour lymph node regions [20].

After the introduction of 3D-CRT, patients were treated using Clinical Target Volumes (CTVs), as outlined on the treatment planning CT scan based on the prechemotherapy tumour volume, taking into account the response to chemotherapy and displacement of normal tissue. The dose was prescribed in accordance with the recommendations of the International Commission on Radiation Units and Measurements for 3D planning, with a 95% isodose coverage of PTV [21,22].

Since the end of 2014, after the ILROG (International Lymphoma Radiation Oncology Group) guidelines [10,19] were introduced, the standard of treatment has become involved-site RT (ISRT) or involved-node RT (INRT), which targets only the sites initially involved with microscopic lymphoma. In addition, RT doses have been deescalated. 

### 2.3. Toxicity and Survival

Acute and late toxicity were scored according to the Radiation Therapy Oncology Group (RTOG) scoring criteria [23]. All patients were seen weekly during radiation treatment. Acute skin, gastrointestinal, and/or haematological toxicities occurring during treatment were extracted from the patients’ charts. 

The side effects of radiation documented six months after the completion of RT were considered late toxicities. The specific late sequels of RT analysed included gastrointestinal dysfunction (including gastritis and ileus), diabetes mellitus (late pancreatic toxicity), renal dysfunction, and secondary tumours (hematologic and solid cancer). 

All times to events (recurrence, death, and late toxicities) were measured from the beginning of the radiation therapy. The cut-off date for patients’ accrual to the study was 31 December 2020, and the cut-off date for follow-up of all events was 31 March 2023, giving a minimum follow-up of more than 2 years. 

Event-free survival (EFS) was defined as the time from the beginning of radiotherapy until failure to respond, tumour progression, or death, whichever occurred first. Overall survival (OS) was defined as the time from the start of radiotherapy to death, irrespective of cause.

### 2.4. Ethical Considerations

This was a retrospective study using anonymized patient data, performed in accordance with the Declaration of Helsinki and all relevant Croatian, EU and international laws and regulations. The study was approved by the Ethics Committees of our Institution

## 3. Results

We identified 140 patients fulfilling the entry criteria (Table 1 and Table 2). The cohort comprised 79 (56%) males and 61 (44%) females with a median age of 57 y (range: 18–85 y). In total, 41 patients were treated using 2D and 99 using 3D techniques. IFRT was used in 69 (49%) patients (40 using 2D RT and 29 using 3D RT), and ISRT/INRT in 71 (51%) (all using 3D RT) (Table 3).

Abdominal regions were irradiated in 79 (56%) patients, abdominal and pelvic in 28 (20%), and pelvic only in 33 (24%). Extranodal sites are listed in Table 4. Patients treated until 2014 received total doses ranging from 12 to 50.4 Gy, with a median dose of 36 Gy, using daily fractions of 175 to 700 cGy 5 times/week depending on treatment intention. Since 2015, the median dose was 30 Gy, ranging from 27 to 48 Gy, using daily fractions between 150 and 300 cGy, 5 times/week depending on the localization and treatment intention.

Overall, the median follow-up was 70 mo (95% CI 58–82); the median follow-up of patients treated with IFRT was 133 mo (95% CI 109–158); and with ISRT, 48 mo (95% CI 39–57).

The 2- and 5-year OS for all included patients in the IFRT and ISRT groups were 79% and 69% vs. 80% and 70%, respectively, (*p* = 0.711). The 2- and 5-year EFS in the IFRT and ISRT groups were 73% and 68% vs. 77% and 70%, respectively, (*p* = 0.575).

**Figure 1:**
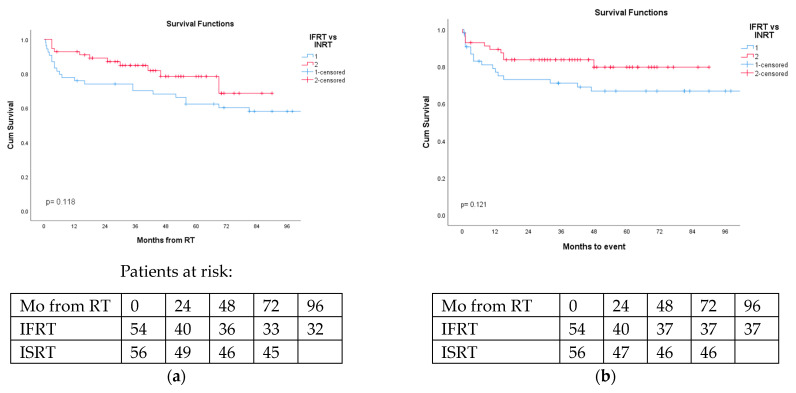
Overall survival and event-free survival of DLBCL patients irradiated via involved-field (IFRT) or involved-site (ISRT) were not statistically different. (**a**) Overall survival of DLBCL NHL patients according to radiation therapy fields (blue-IFRT vs. red-ISRT); *p* = 0.118. (**b**) Event-free survival of DLBCL NHL patients according to radiation therapy fields (blue-IFRT vs. red-ISRT); *p* = 0.121.

The 2- and 5-year OS for DLBCL patients in the IFRT and ISRT groups were 74% and 62% vs. 87% and 78%, respectively, (*p* = 0.118). The 2- and 5-year EFS in the IFRT and ISRT groups were 73% and 69% vs. 84% and 80%, respectively, (*p* = 0.121) (Figure 1a,b).

In a univariate analysis for DLBCL patients, age, gender, number of treatment lines, and treatment intent were statistically significant prognostic factors for OS (Table 5). In a multivariate analysis, including the sex, age, stage of disease, extranodal location, number of chemotherapy lines, treatment intent, RT field, and method of irradiation, only age and sex were significantly associated with overall survival (Table 6). 

The locoregional control rate in the infradiaphragmal regions was 70% (98 of 140) for the entire group. In patients treated with IFRT, the locoregional control was 67%, and in those treated with ISRT, 73%. There were statistically significantly (*p* < 0.001) more relapses in patients receiving salvage than adjuvant RT (63% vs. 21%, respectively). The probability of relapse was not related to the site of radiation (abdomen, pelvis), size of radiation field (IFRT vs. ISRT), or extranodal presentation of the lymphoma.

Acute side effects occurred in 43 (31%) patients. They were more frequent in the IFRT group (34 (39%) patients) than in the ISRT group (9 (13%) patients), *p* < 0.01. Gastrointestinal side effects, such as nausea, vomiting, and diarrhoea, were most frequent and occurred in 19 (28%) patients in the IFRT group and 5 (7%) patients in the ISRT group (Table 7). There was no grade 4 or 5 toxicity. 

Late side effects occurred in 13 of 103 (13%) patients followed for more than 2 years, 9 of 56 (16%) followed for more than 5 years, and 6 of 23 (26%) followed for more than 10 years. Four patients developed renal failure; three, gastrointestinal motility problems (including one emergency surgery for ileus); two, diabetes mellitus; and six, secondary cancers (3 colon, 1 rectal, 1 pancreatic, and 1 acute myeloblastic leukaemia). Two patients had two different late side effects. Late toxicities occurred more often (10/57—17.5%) in the IFRT group than in the ISRT group of patients (3/62—5%), respectively, (*p* = 0.026).

## 4. Discussion

RT is a well-established treatment option for the management of aggressive non-Hodgkin lymphoma (aNHL), which encompasses different histological and clinical sub-types. It is an important component of multimodal therapy for many patients and remains the most effective single modality for local disease control [10,24]. The evidence from retrospective and randomised trials supports a significant benefit of RT in enhancing local disease control, disease-free survival and, ultimately, overall survival even after modern multiagent chemotherapy [3,7,25,26,27,28,29]. These findings underscore the importance of considering RT as a valuable adjunctive treatment modality. However, despite this positive finding, the lack of randomized trials demonstrating the efficacy of consolidative RT in treating bulky advanced stages, especially in the rituximab and PET-CT eras, emphasizes the need for further research and exploration in this patient population [30,31,32,33]. This paper aims to shed light on the intriguing and underexplored aspects of infradiaphragmatic radiotherapy in the management of aggressive NHL. We aimed to evaluate the results of the evolution in our clinical practice, particularly focusing on toxicity and the efficacy of IF and INRT/ISRT in a “real-life” setting. 

The patient population included in this study was heterogeneous in terms of histological diagnosis, indication for radiotherapy, and other treatments received. Additionally, it is important to note that this study is retrospective and non-randomized in nature, which may limit the generalizability of the findings to specific clinical settings. However, the majority of our patients had diffuse large B-cell lymphoma (DLBCL) and received state-of-the-art immunochemotherapy, R-CHOP (rituximab, cyclophosphamide, doxorubicin, vincristine, steroids) or similar, as front-line treatment. The survival rates in our study (68% at 5 years) are consistent with lymphoma outcomes. According to the National Cancer Institute data from 2013 and 2019, the 5-year overall survival rate for patients with diffuse large B-cell lymphoma (DLBCL) is 64.7% [34]. 

Regarding RT doses, Hoskin et al. suggested in 2013 that there is increasing evidence that traditional doses of RT are higher than necessary for disease control in NHL [13]. Nowadays, doses of up to 30 Gy for aNHL in the adjuvant setting are recommended [10,35]. Our experience is in accordance with these recommendations. We have not seen a reduction in efficacy with the reduction in total RT doses from the previously standard 40 Gy to the currently recommended 30 Gy.

In terms of irradiated volumes, modern radiation fields are designed to exclusively irradiate the initially involved lymph nodes encompassing their initial volume. In some cases, radiation fields are slightly modified to avoid the unnecessary irradiation of muscles or organs at risk [10,31]. Our analysis demonstrates that reducing irradiation volumes to ISRT decreases both acute and chronic toxicities. This finding is consistent with published data [36,37,38]. Proton therapy is another possibility for minimizing the sequelae of irradiation [39,40,41,42], but there are no reports on the use of this technique in infradiaphragmal RT. 

Regarding safety, acute side effects required an interruption of RT occurred in 6% of patients (grade 3 toxicity). Gastrointestinal (GI) side effects were most frequent, in accordance with other studies on abdominal region irradiation [43,44,45,46]. With increasingly effective curative treatment regimens, there is a growing concern about the late side effects of treatment and the quality of “survivorship”. In our study, the frequency of late side effects 5 years after the irradiation of the infradiaphragmal areas was 16%, increasing to 26% after 10 years, which is consistent with published reports [47,48,49]. Secondary tumours predominantly occurred in irradiated areas, with a median latency period of 90 months (more than 7 years) post irradiation. Notably, all but one patient with secondary tumours were irradiated via involved-field RT.

The risk of developing secondary malignancies following radiotherapy is the subject of numerous controversies. Patients undergoing RT have an increased risk of developing other carcinomas due to lifestyle and genetic predispositions [50,51], which may be more pronounced than the radiation risk itself. Our findings echo the challenges highlighted in the literature, where smaller studies from single institutions often failed to detect an elevated risk for carcinoma development after RT [52]. However, larger-scale research involving more patients has managed to show a slight but statistically significant increase in the risk of carcinoma development after radiotherapeutic treatment [53,54]. Pelvic area irradiation for primary tumours of the cervix, prostate, or testicles introduces an elevated risk of developing secondary carcinomas in various organs, which is consistent with previous reports [55,56,57,58]. These tumours, induced via radiotherapy, mostly arise with a latency of at least 5–10 years [59,60]

The toxicity of RT can be reduced through dose reductions, volume reductions, and the optimization of using IMRT (intensity-modulated radiotherapy). IMRT and VMAT (volumetric modulated arc therapy) are more effective than 3D-CRT in terms of target coverage, dose homogeneity, and reducing toxicity to normal organs [61,62,63]. IMRT/VMAT techniques were not available at the time at our institution. However, most recurrences in patients treated for aNHL occur in the sites of initial involvement, and RT is highly effective at reducing subsequent local recurrences [7,27]. Therefore, it is crucial to optimize the delivery of RT to maintain high rates of long-term local control while minimizing the radiation exposure of surrounding normal tissues. 

In conclusion, our study shows that, in a real-life setting, the modern RT of infradiaphragmal fields in aNHL, with a reduction in the total dose to 30 Gy and irradiation volume of IS/INRT, reduces early and late side effects while maintaining efficacy.

## 5. Conclusions

Our study, conducted in a real-life clinical setting, provides compelling evidence for the efficacy and improved tolerability of modern radiotherapy in treating infradiaphragmal aggressive NHL. By implementing a reduced total dose of 30 Gy and limiting the irradiation volume of the involved-site radiotherapy, our findings consistently demonstrate a significant reduction in both early and late side effects without compromising treatment efficacy. 

## Figures and Tables

**Table 1:** Patient and clinical characteristics.

Characteristics	No.	%
*Sex*		
Male	79	56
Female	61	44
*Age*		
<60 years	89	64
≥60 years	51	36
*Stage of the disease*		
I	16	11
II	25	18
III	33	24
IV	66	47
*Number of treatment lines*		
0–1	106	76
≥2	34	24
*Irradiation site*		
Abdomen only	79	56
Abdomen and pelvis	28	20
Pelvis	33	24
*Method of irradiation*		
2D	41	26
3D	99	74
*Radiotherapy fields*		
IFRT	69	49
ISRT	71	51
*Treatment intent*		
adjuvant	48	34
consolidation	62	44
salvage	30	21

**Table 2:** Lymphoma type in infradiaphragmal region.

	No.	%
Burkitt	3	2
DLBCL	110	79
FL, grade 3b	9	6
Mantle cell	8	6
T-NHL	4	3
Transformed indolent	6	4

**Table 3:** Cross-table for the relationship between 2D-RT and 3D-RT and IFRT and ISRT.

	2D-RT	3D-RT	Total
IFRT	41	28	69
ISRT	0	71	71
Total	41	99	140

**Table 4:** Distribution of extranodal NHL according to radiotherapy fields.

EXTRANODAL	No. of Patients	
	IFRT	ISRT
gastric	9	11
testis	2	1
vagina, cervix, uterus	0	3
prostate	0	1
kidney and adrenal	0	2
muscle and bone	7	1
skin	1	0
small intestine	1	0

**Table 5:** Univariate analysis of overall survival in DLBCL patients. ASCT—autologous stem cell transplantation.

OS			
Parameters	HR (95% CI)	5-Year OS	*p*
**Sex**	0.5 (0.2–1)		0.045
m		60%	
f		81%	
**Age**	3.4 (1.7–6.8)		0.001
<60		81%	
≥60		50%	
**Stage of disease**	0.8 (0.4–1.6)		0.570
I and II		77%	
III and IV		65%	
**No. of treatment lines**	3.1 (1.5–6.3)		0.002
1		76%	
≥1		40%	
**Extranodal**	0.9 (0.5–2.1)		0.916
Yes		69%	
No		71%	
**RT fields**	0.6 (0.3–1.2)		0.125
IFRT		62%	
ISRT		78%	
**Treatment intent**	3.6 (0.3–7.7)		0.001
Adjuvant/consolidation		75%	
Salvage		36%	
**ASCT before RT**	1.2 (0.4–3.3)		0.762
Yes		69%	
No		68%	
**Method of irradiation**	0.8 (0.4–1.6)		0.507
2D		63%	
3D		71%	

**Table 6:** Multivariate analysis for the prediction of overall survival in DLBCL patients.

Overall Survival	*p* Value	HR (95% CI)
Sex (m vs. f)	0.032	0.4 (0.2–0.9)
Age (≤60 years vs. >60 years)	0.001	3.9 (1.7–8.7)
Stage of disease (I/II vs. III/IV)	0.957	0.9 (0.4–2.2)
Extranodal disease	0.061	0.3 (0.1–1.0)
RT fields (IFRT vs. INRT)	0.343	0.7 (0.3–1.5)
No. of chemotherapy lines (1 vs. ≥1)	0.312	2.3 (0.5–11.3)
Treatment intent	0.147	3.6 (0.6–19.7)
Method of irradiation (2D vs. 3D)	0.292	0.6 (0.2–1.6)

**Table 7:** Acute side effects in the IFRT and ISRT fields.

	IFRT		ISRT	
	No	%	No	%
Grade I	25	36	9	13
Grade II	5	7	0	0
Grade III	4	6	0	0
Haematological	10	14	2	3
Gastrointestinal	19	28	5	7
Other (pain)	7	8	2	2

## Data Availability

The data can be shared up on request.
